# Supplementary material for: Neurotrophic Effects of Foeniculum vulgare Ethanol Extracts on Hippocampal Neurons: Role of Anethole in Neurite Outgrowth and Synaptic Development
Source: Int J Mol Sci. 2024 Nov 26;25(23):12701. doi: 10.3390/ijms252312701 (PMC11641539; doi:10.3390/ijms252312701)
Supplement: Supplementary file 1 [file ijms-25-12701-s001.zip › ijms-3268385-supplementary.pdf]

Supplementary File-1

Neurotrophic Effects of *Foeniculum vulgare* Ethanol Extracts on Hippocampal Neurons: Role of Anethole in Neurite Outgrowth and Synaptic Development

Sarmin Ummey Habiba [1], Ho Jin Choi [1,2], Yeasmin Akter Munni [1,3], In-Jun Yang [3], Md. Nazmul Haque [4] and Il Soo Moon [1,*]

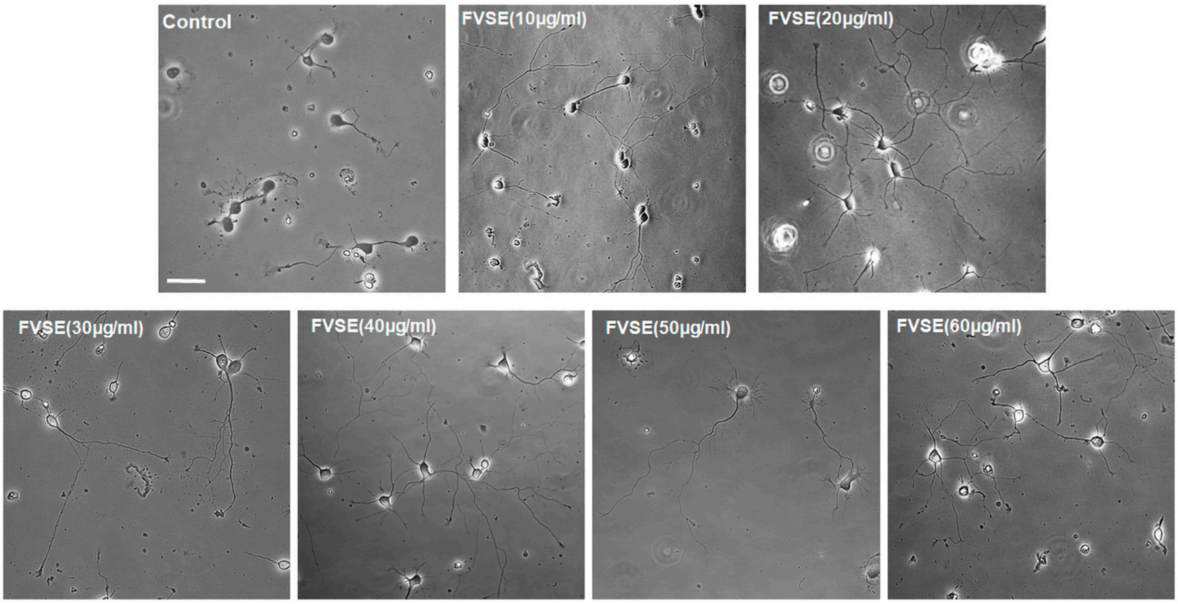

Figure S1: FVSE promotes neuritogenesis in a dose-dependent manner. Representative phase-contrast images showing neurite outgrowth of FVSE-treated neurons on DIV3 in a dose-dependent manner; scale bar: 50 μm.

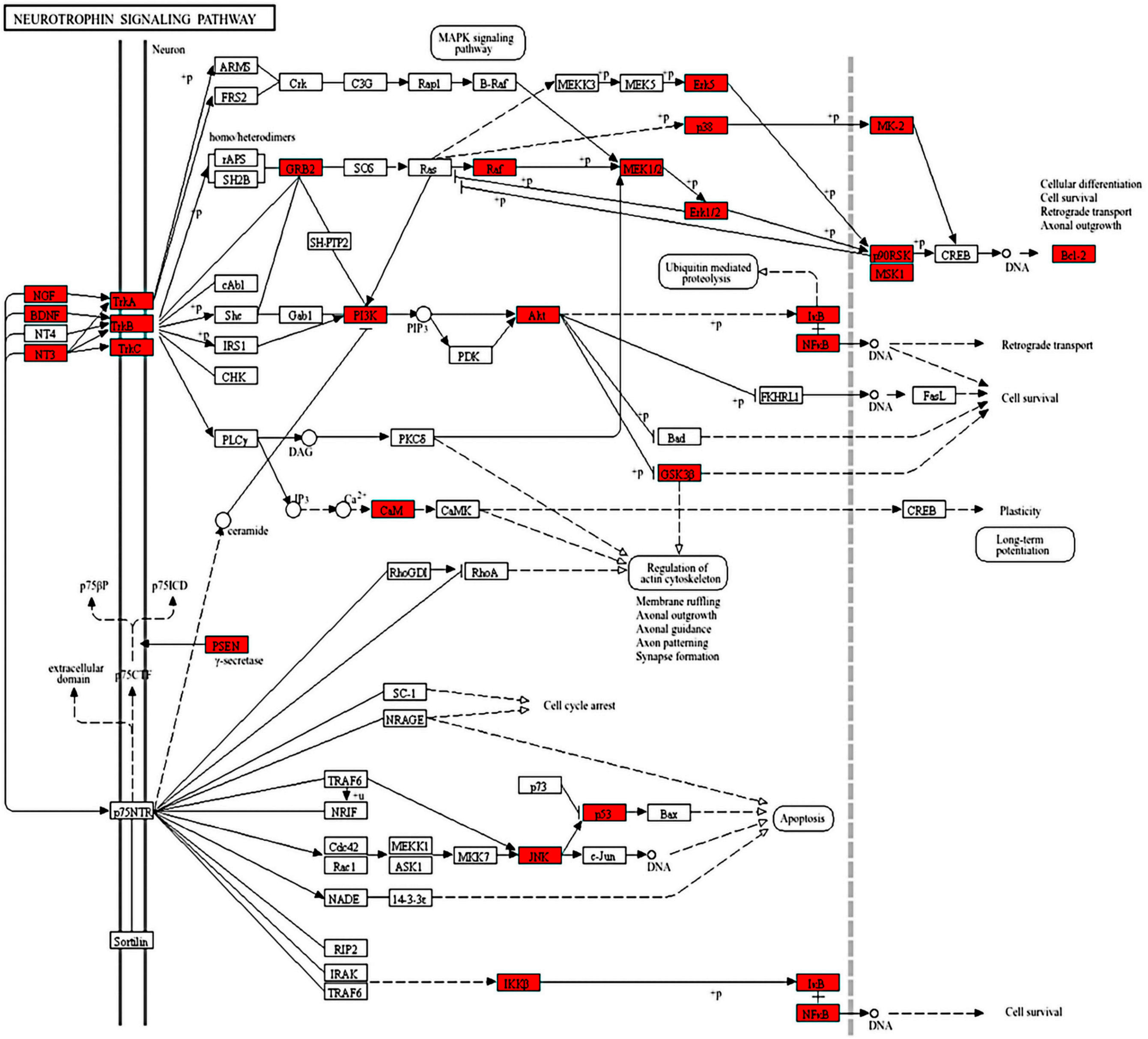

Figure S2: The neurotrophin signaling pathway influenced by bioactive components of FVSE. KEGG pathway analysis reveals the involvement of key genes modulated by the bioactive compounds Anethole, silane ethoxy(dimethylphenyl)-, and para-anisaldehyde diethyl acetal, all of which are present in FVSE seed extract. The red boxes in the figure modulated the genes affected by these components.

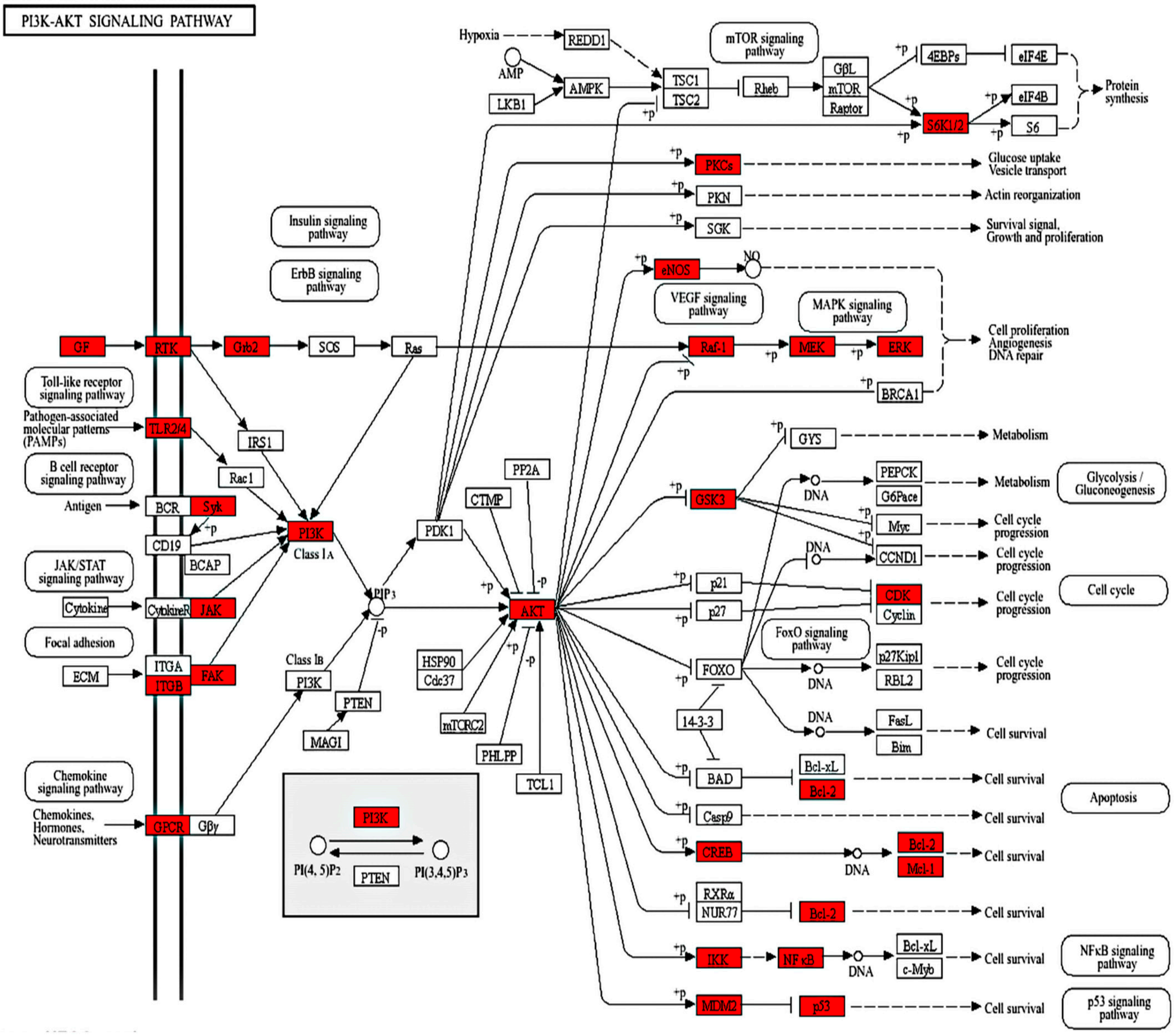

Figure S3: The diagram illustrates the PI3K-Akt signaling pathway, which is essential for the neuroprotective effects of FVSE treatment. This pathway encompasses proteins and molecular interactions that are vital for cell survival, growth, metabolism, and inhibition of apoptosis. Key nodes marked in red indicate components upregulated or modulated by FVSE, as identified in our study. Activation of the PI3K-Akt pathway by FVSE potentially enhances neuronal survival by promoting anti-apoptotic signals and neuroplasticity, which are crucial for countering neurodegenerative processes in conditions such as Alzheimer's and Parkinson's diseases.

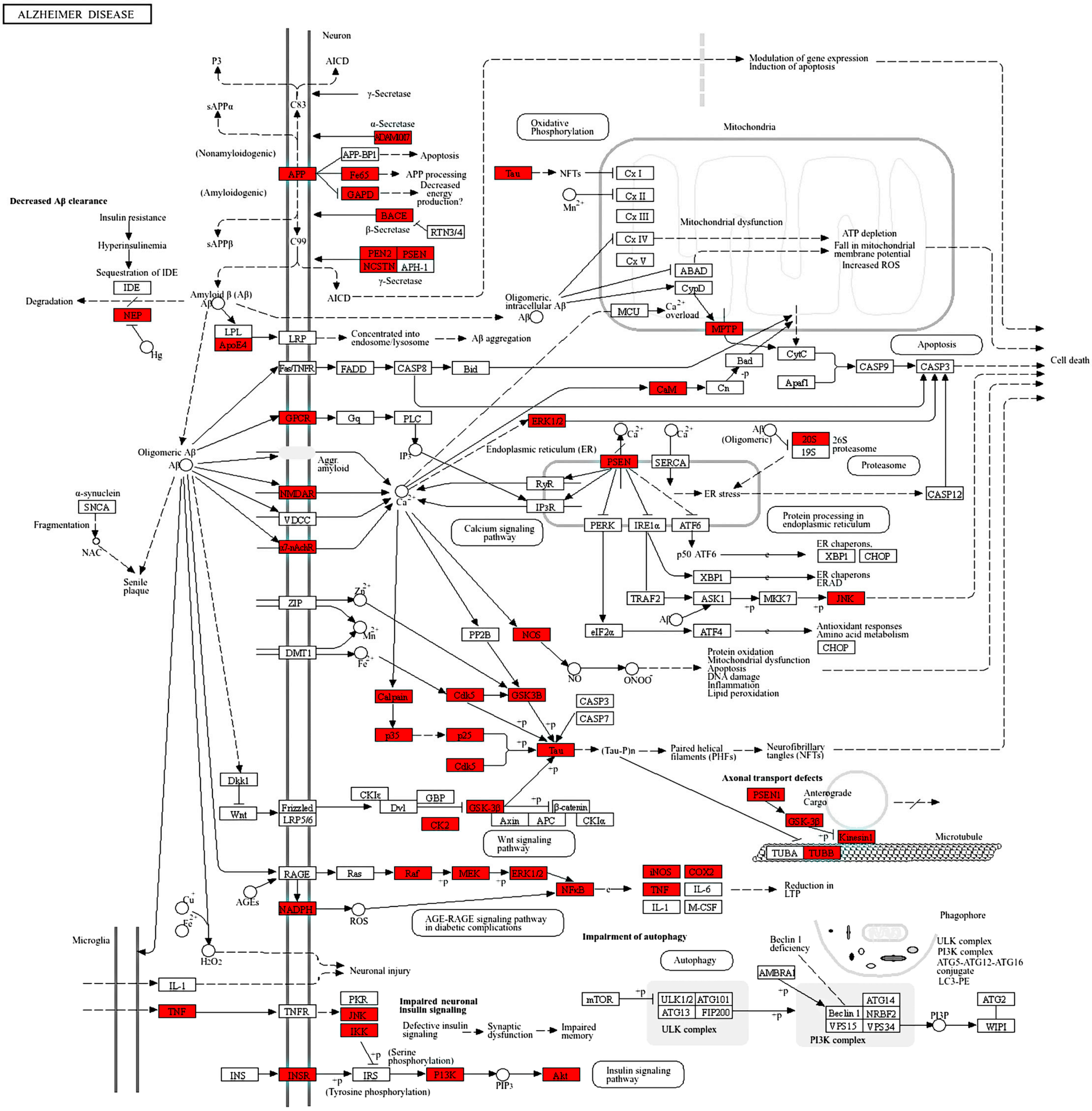

Figure S4: The diagram illustrates the Alzheimer's disease pathway, highlighting the crucial proteins and processes involved in disease progression. Red proteins indicate potential modulatory targets of FVSE, which may influence neuroprotective mechanisms by interacting with pathways related to amyloid-beta processing, tau protein aggregation, oxidative stress, and neuronal apoptosis. By targeting these pathways, FVSE may reduce neurodegeneration and improve cognitive function.

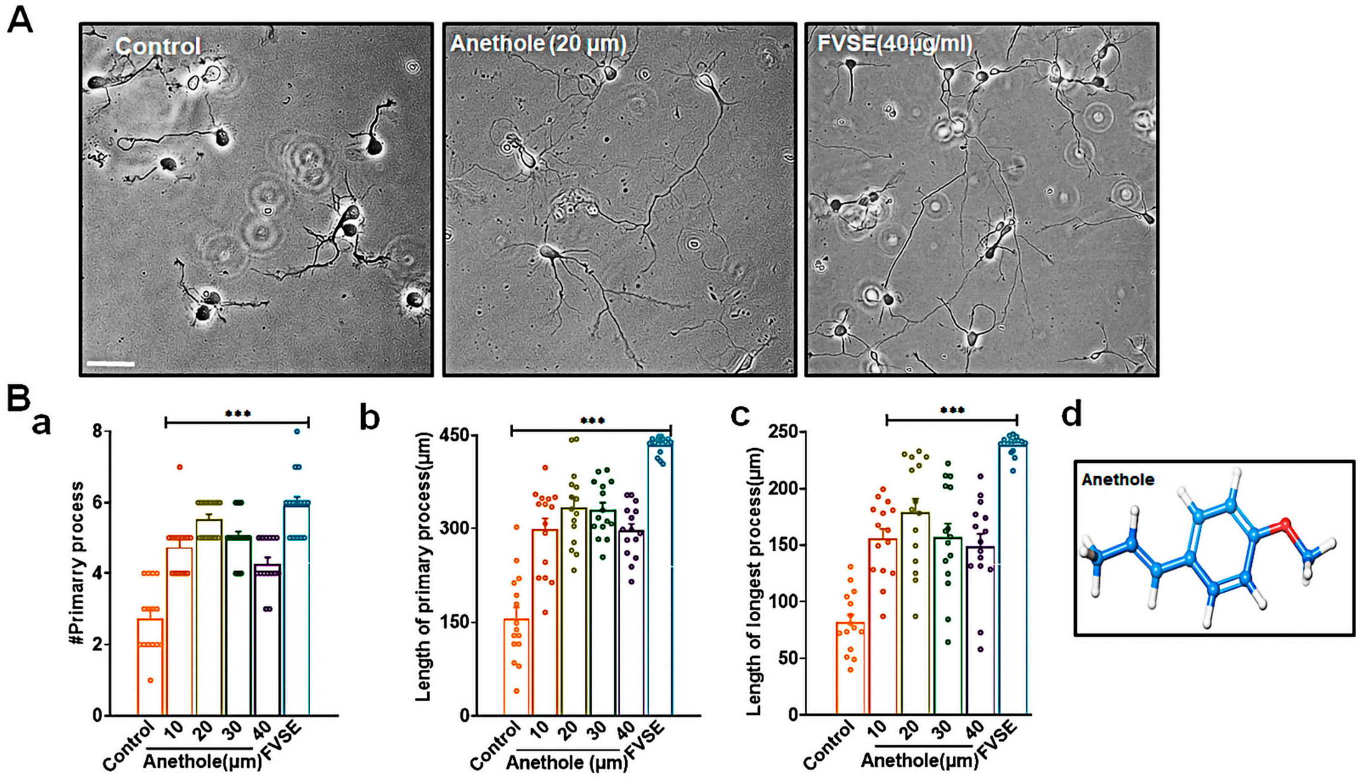

Figure S5: Anethole promotes neurite outgrowth. (A) Representative bright-field images of primary hippocampal neurons treated with Control, Anethole (20 µM), or FVSE (40 µg/mL) showing enhanced neurite outgrowth. (B) Quantitative analysis of neurite outgrowth: (a) number of primary processes per neuron, (b) length of primary processes, and (c) length of the longest process. Data are presented as the mean ± standard error of the mean (S.E.M.) from three independent experiments ($n = 5$), with 15 neurons analyzed per condition. Statistical significance was determined using ANOVA ($p < 0.001$). (d) 3D molecular structure of Anethole. Scale bar, 50 µm.
